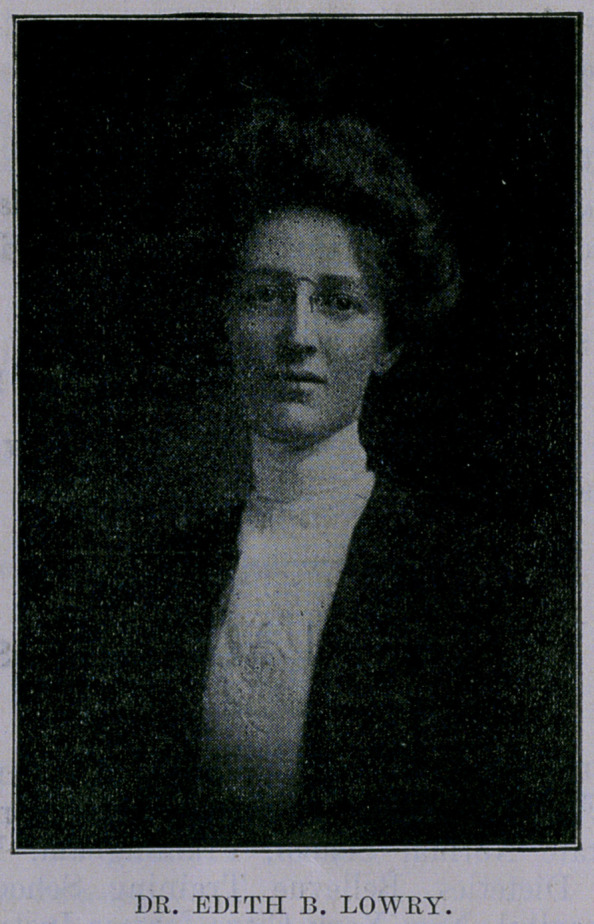# Books and Magazines

**Published:** 1911-04

**Authors:** 


### Books and Magazines.

PRACTICAL DIETETICS—WITH REFERENCE TO DIET IN DISEASE. By Alida Frances Pattee, Graduate, Department of Household of Arts, State Normal School, Framingham, Mass.; late Instructor in Dietetics, Bellevue Training Schools for Nurses, Bellevue Hospital, New York City; former Instructor at Mount Sinai, Hahnemann, and the Flower Hospital Training Schools for Nurses, New York City; Lakeside, St. Mary's, Trinity and Wisconsin Training Schools for Nurses, Milwaukee, Wis.; St. Joseph's Hospital, Chicago, Ill.; St. Vincent de Paul Hospital, Brockville, Ontario, Canada. Sixth edition. Enlarged and revised to meet the exact requirements of the various State Boards of Examiners of Nurses. 12mo. Cloth. 550 pages. Price, $1.50 net, postpaid. A. F. Pattee, Publisher, 134 S. First Avenue, Mt. Vernon, New York.

EXTRACT FROM PREFACE TO SIXTH EDITION.

Advantage has been taken of the demand for a sixth edition to revise the entire book, and incorporate the latest results of research in dietetics in a concise form.

The contents of the book have been arranged to correspond to the exact requirements of the various State Boards of Examiners of Nurses. This will materially aid the nurse in preparing for her State examination.

To meet the growing tendency of the physician to prescribe the exact food value of a diet, the energy values of each recipe is given and the exact food value of a large number of common foods, so that no calculations are necessary.

DR. EDITH B. LOWRY.

Dr. Edith B. Lowry, author of two notable books reviewed in this issue.

---

CONFIDENCES.—TALKS WITH A YOUNG GIRL CONCERNING HERSELF. By Edith B. Lowry, M. D. A book explaining the origin and development of life in language intelligible to young girls. The author, who is a physician of wide experience and a pleasing writer has very delicately and adequately treated this important subject. The future health and happiness of every girl demands that she receive when approaching adolescence an intelligent presentation of the vital life processes, and this book will be an invaluable aid to parents and teachers in attaining that object. Neatly bound in cloth. 16mo. Price, postpaid, 50 cents net. For sale by all booksellers or sent postpaid by the publishers. Forbes & Company, 325 Dearborn Street, Chicago.

THE AUTHOR'S PREFACE TO "CONFIDENCES."

"No one can come in contact with children and young people without feeling the need of a united effort on the part of the parents, physicians and teachers to lessen the immoral tendencies, with their degrading effects, to which the present generation is subjected. Knowledge of the right sort will prevent many wrecked lives. Ignorance as to facts and to the best manner of presenting them prevents many a parent from daring to trespass upon such sacred ground, and the instruction is postponed from day to day until it is too late.

"With the desire to aid mothers in giving the necessary instruction to their daughters, this little book has been written. The author has tried to tell in suitable language the facts that should be known by every girl from ten to fourteen years of age. The book is of such a character that it may be placed in the hands of the young girl, but, better still, it may be read aloud by the mother to her daughter. It is hoped this book will form the basis of a closer intimacy between mother and daughter, and that the knowledge herein set forth will forestall that which might be given in an entirely different spirit by the girl's companions."

Dr. Charles W. Eliot, former President of Harvard University, recently said: "The subject of reproduction and sexual hygiene should be more generally presented to young people by parents and teachers. I am convinced that the policy of silence has failed disastrously."

---

TRUTHS.—TALKS WITH A BOY CONCERNING HIMSELF. By E. B. Lowry, M. D., Author of "Confidences." A book containing the simple truths of life development and sex which should be given to every boy approaching manhood. His future welfare demands it. This is the first book to adequately and delicately present these truths in language intelligibly to boys from ten to fourteen years of age. Parents, teachers and physicians will find it a needed and helpful book of inestimable value. Neatly bound in cloth. 16mo. Price, 50 cents net, postage 5 cents. Forbes & Company, 325 Dearborn Street, Chicago.

FROM THE AUTHOR'S PREFACE TO "TRUTHS."

Dr. Charles W. Eliot, former President of Harvard University, recently said: "The subject of reproduction and sexual hygiene should be more generally presented to young people by parents and teachers. I am convinced that the policy of silence has failed disastrously."

How to present this knowledge depends upon the age, environment and circumstances. With the very young child, who lives almost entirely in a world of imagination, the poetical fancies often can be used to good advantage. But when the boy has reached a school age and associates with older boys, things begin to assume more natural proportions and the world takes on a more real aspect. Then it is the boy wants more material explanations, demands practical truths. A man can ill-afford to allow vulgar representations of these most sacred truths to be given to his boy by his companions, but he may rest assured they will be, and the boy will listen unless this has been forestalled by knowledge given by a wise parent.

With the desire to help fathers and mothers in their work of protecting their sons this book has been written.

---

MALARIA AND ITS MANIFESTATIONS—"With the most thorough and exhaustive methods of treatment of any work of its kind on the subject." By J. H. McCurry, M. D., Grubbs, Ark. Press of S. C. Tod & Co., Memphis. 1910. Cloth. $1.50.

This little book is a complete epitome—or rather a digest of all that is known on the subject of malaria, its diagnosis and treatment. By the by, is it not about time to give this disease a rational name, since we know that it is not caused by "bad air"?

Quiz Compends on Gynecology. By Wm. Hughes Wells, M. D., Associate in Obstetrics in the Jefferson Medical College; Assistant Obstetrician in the Jefferson Medical College Hospital, etc. Fourth edition, revised and enlarged. 153 illustrations. Philadelphia. 1911. P. Blakiston's Sons & Co. Price, $1.00.

This little manual is very popular with students and is well known. Its purpose is sufficiently set forth in the title—"Cram for exam."

A MANUAL OF CYSTOSCOPY. By J. Bentley Squier, M. D., Professor of Genito-Urinary Surgery, New York Post-Graduate Medical School and Hospital; Surgeon to Work House Hospital and Home for Aged and Infirm, Department of Charities and Corrections, New York; Consulting Surgeon, New York Neurological Hospital; Fellow American Association of Genito-Urinary Surgeons; Fellow New York Academy of Medicine, and Henry G. Bugbee, M. D., Instructor in Genito-Urinary Surgery New York Post-Graduate Medical School and Hospital; formerly Surgeon in Chief, Vassar Brothers' Hospital, Poughkeepsie; Fellow New York Academy of Medicine. With twenty-six original plates, eighteen of which are colored. Octavo, flexible leather, $3.00 net. Sent prepaid on receipt of price. Paul B. Hoeber, Medical Publisher, Bookseller and Importer, 69 East Fifty-ninth Street, New York. 1911.

This book has been written in response to the repeated applications from our students for a short practical work on cystoscopy.

The statements are concise, and the technique described in detail is that which we have found most satisfactory after a thorough trial of other methods. While the essentials of a working knowledge of the instruments are set forth, the book has been kept within small compass.

The feature of anatomical diagrams accompanying the principal bladder landmarks, as depicted through the cystoscope, will be of great value to the beginner, and the colored plates of intravesicle lesions are particularly accurate, having been produced under the direct observation of the artist.

## Figures and Tables

**Figure f1:**